# Editorial: Metabolic regulation of gamete function in health and disease

**DOI:** 10.3389/fcell.2022.1040708

**Published:** 2022-10-19

**Authors:** Débora J. Cohen, Vanina G. Da Ros, Nicolas G. Brukman, Farners Amargant

**Affiliations:** ^1^ Laboratorio de Mecanismos Moleculares de la Fertilización, Instituto de Biología y Medicina Experimental (IByME-CONICET), Buenos Aires, Argentina; ^2^ Department of Biology, Technion- Israel Institute of Technology, Haifa, Israel; ^3^ Department of Obstetrics and Gynecology, Feinberg School of Medicine, Northwestern University, Chicago, IL, United States

**Keywords:** sperm, egg, gametes, testis, ovary, metabolism

Fertilization is a key process in which two different cell types, sperm and eggs, interact and fuse to merge their haploid genomes and initiate the development of a new individual. Sperm and eggs are highly differentiated cells, and each gamete type fulfills its specific function in different ways. Whereas sperm must leave the body in which they were produced to swim until they meet the female gamete, oocytes must prepare to support the early stages of embryonic development. In both cases, the correct functionality of each gamete could be influenced not only by its own metabolic activity but also by the reproductive and systemic metabolic environment of the individual. In view of this, the goal of this Research Topic was to understand how the systemic or cellular metabolic networks can influence the male and female reproductive function.

The final Research Topic has seven original research articles and one review from 46 authors from nine different countries. These contributions cover critical aspects of metabolic regulation of gonads, gametes and embryos by using different approaches and models, including human, mouse and bovine.

In the group of the gonad related articles, Pelletier et al. explored the hypothesis that cholesterol homeostasis is regulated by local factors in the testis. In particular, by using a battery of genetically modified mouse models, they found that PCSK9, an enzyme which contributes to cholesterol homeostasis, is important in both cholesterol and glucose homeostasis as well as in the immunotolerance in the testis. In the ovary, Xiang et al. reported that chronic unpredictable mild stress induces alterations in folliculogenesis and a significant reduction in estradiol and anti-mullerian hormone levels. Additionally, they found that lipid homeostasis is dysregulated in the depression-like mice suggesting a potential link between stress, lipid homeostasis and ovarian function.

In the field of gametes, four articles covered different aspects of sperm metabolic regulation. Marín-Briggiler et al. tested whether temporary energy restriction regulates sperm capacitation-related events in humans. Interestingly, they reported that human sperm remain motile after starvation due to mitochondrial use of endogenous metabolites. This is in contrast to what has been reported in mice, as sperm stop moving upon starvation. Overall this work suggests that mouse and human sperm might have intrinsic metabolic variations.

Irigoyen et al. focused on improving the clinical evaluation of male infertility by integrating mitochondrial functional studies to the routine semen analysis. To this end, they found important correlations between sperm mitochondrial function (i.e., oxygen consumption and ROS production) and sperm parameters (i.e., motility and morphology). In addition, they developed appropriate assays to evaluate routinely these parameters which could help to explain some cases of infertility.

The work by Tourmente et al. explored changes in glycolytic and respiratory parameters of murine sperm during capacitation using an extracellular flux analyser in freely moving cells. Results show that capacitation promotes a shift in the usage ratio of the metabolic pathways, from oxidative to glycolytic, without affecting the ATP consumption rate, probably as a mechanism to ensure the ATP supply in the distal flagellar regions required during or after capacitation.

Numata et al. discovered an unexpected link between the function of the testis specific ion transporter, Na, K-ATPase α4, which is known to be essential for sperm motility, and the cell energetics. They found that sperm deficient in this channel have widespread consequences on energy production reflected in an impaired glycolytic activity, mitochondrial function, increased production of reactive oxygen species and more, setting the bases of a new mechanism for the regulation of sperm function.

Regarding embryo development, Aardema et al. focused on the low cryotolerance of *in vitro* produced embryos. They proposed that the addition of fatty acid complexes positively affects the cryopreservation properties of *in vitro* produced bovine blastocysts. Their results could contribute to improving embryo transfer results not only in husbandry species but also in infertile human couples.

Finally, a Review article by Arias et al. explores the relevance of cholesterol for oocyte biology and female fertility. Based on existing evidence they suggest an homeostatic mechanism that involves high-density lipoproteins (HDL) within the follicular fluid and regulates the cholesterol levels in the oocyte, with potential implications for female fertility.

Altogether, the papers included in this Research Topic highlight the relevance of metabolism on the function of reproductive systems and gametes. Regardless of the mysteries that are pending to be explored, the evidence gathered in this Research Topic has the potential of significantly improving the diagnosis and treatment of metabolic-related infertility as well as boosting animal biotechnology.

